# Supplementary material for: Effects of fenbendazole on fecal microbiome in BPH/5 mice, a model of hypertension and obesity, a brief report
Source: PLoS One. 2023 Jun 9;18(6):e0287145. doi: 10.1371/journal.pone.0287145 (PMC10256194; doi:10.1371/journal.pone.0287145)
Supplement: Attachment — Submitted filename: Comments_plosOne_FBZ_2023.docx [file pone.0287145.s001.docx]

**Effects of fenbendazole on fecal microbiome in BPH/5 mice, a model of hypertension and obesity**

Beckers et al

Thank you for reviewers’ comments, see red lined below. We have rearranged the discussion per editor’s suggestion to better link results to discussion points.

Please use the space provided to explain your answers to the questions above. You may also include additional comments for the author, including concerns about dual publication, research ethics, or publication ethics. (Please upload your review as an attachment if it exceeds 20,000 characters)

Reviewer #1: Abstract: line 42, define FBZ.

Thank you, we have corrected the mistake and added the definition on line 31.

Please clearly mention which comparisons (gender, medication, before/after medication, obesity) are you targeting. Each result line in mentioned for a different comparison. Please give

A sentence was added to the abstract line 36-38 to aid in clarification. The main purpose of the study was to analyze fecal microbiome communities before and after medication, but it was noted that the alteration were made in a sex dependent manner.

Give Bray-Curtis dissimilarity p value. The difference is only between male and female which means treatment has no effect. Please give each p values. Results are not coherent with the conclusions.

P values have been added to the abstract and results edited, lines 39-44

I wouldn’t say Lactobacillus as a marker of healthy gut. Lactobacillus at genus level has more complex role.

Removed from the abstract

Methods:
What we mean by Adult (8 weeks-6 months of age)?

Thank you, verbiage was added to clarify.

Line 86 to 91: break in smaller sentences.

Corrected, thank you.

Line 102, 103: were not are

Corrected.

Line 107 : exiting the room, respectively

Added, thank you.

Line 109: Samples collection:

Corrected, thank you.

Line 110: individually in sterile empty cages

Corrected, thank you.

Line 101: were placed in sterile tubes

Corrected, thank you.

Line 129: Microbial community

Corrected, thank you.

Changes at the phyla level in BPH/5 mice with Fenbendazole treatment
Herein just mention treatment effects (FBZ). Or if you want to add gender as well, make a crystal clear difference from the start.
Only mention changes with p value < 0.05 or elsewhere mention p value. Result sentences are not written properly, and each sentence has multiple results with no clear reflection. Sometimes past and present are mixed (164-165).

P values have been added to results and result section has been edited to make things clear.

Either figures are not clear, or p values are not mentioned. For figure 2. C, D, and E, use the same headings. As I can see that the post-male has larger bell size, this may lead to non-significant results. Lines 181 to 184: reflect that the effect is due to treatment which contradicts with the conclusions. Overall, I believe that we shall rewrite results with more coherent text and figures.

Thank you for pointing out this flaw. Titles are corrected on all figures to the same headings and p-values have been added to each figure as well.

179: dissimilarity

Corrected, thank you.

Discussion:
Line 203: What we mean by institution?

The intended meaning was location of where those mice were housed. Corrected in text, thank you.

Figure 2 A and B; also use the similar titles. In PCoA I can see that post treatment clustering is poor, which needs to be justified.

Titles have been edited to be similar. Clustering is poor due to the hypothesis that the treatment affected the males more greatly cause a greater community change in the post treatment samples.

Reviewer #2: You should acknowledge as a limitation that although you found some differences in microbial frequency, you did not evaluate effects on blood pressure or obesity, which are key features of the animal model you studied. Also, 16S rRNA testing is relatively insensitive compared to more complete genomic analyses.

Thank you for the input. This is a good point and has been added to the discussion on lines 240-243.
