# Supplementary material for: Effects of fenbendazole on fecal microbiome in BPH/5 mice, a model of hypertension and obesity, a brief report
Source: PLoS One. 2023 Jun 9;18(6):e0287145. doi: 10.1371/journal.pone.0287145 (PMC10256194; doi:10.1371/journal.pone.0287145)
Supplement: Attachment — Submitted filename: Commets_v2_plosone_FBZ_2023 (1).docx [file pone.0287145.s002.docx]

**Effects of fenbendazole on fecal microbiome in BPH/5 mice, a model of hypertension and obesity**

Beckers et al

Thank you for reviewers’ comments, see red lined below. We have rearranged the discussion per editor’s suggestion to better link results to discussion points.

Please use the space provided to explain your answers to the questions above. You may also include additional comments for the author, including concerns about dual publication, research ethics, or publication ethics. (Please upload your review as an attachment if it exceeds 20,000 characters)

Reviewer #3: The manuscript by Beckers et al. addresses the effect of FBZ anti-parasitic treatment on stool microbiota in a mouse model of hypertension and obesity. This manuscript is well written and the analyses are straightforward. I reviewed the R1 version of the manuscript which was a response to the comments from 2 other reviewers. My comments do not take into account these earlier comments and I leave it to the editor which of my comments should be addressed. I do have a few comments:

One interesting aspect was not addressed in the discussion. These mice are inbred and are housed and fed in a very controlled environment. Therefore, I would expect (although I am not very experienced in the laboratory mouse stool microbiome) that inter-individual differences are not as high as presented in Fig. 1. If the number of words allows, I would like to see some comments on that in the discussion.

Even though these mice are inbred and were housed in the same environment, some individual variation is noted on the bar graph of Figure 1, but nothing was significantly different when the individuals were used as the random effect in the 2-way ANOVA. The only thing to note of significance was the phyla Actinobacteria and Verrucomicrobia when assessing the time points pre and post, which is presented in Figure 2.

Another interesting aspect that was not clearly addressed. This is longitudinal data. The analyses can make use of these differences before and after treatment for each animal. That can be done for alphas as well as single genera. Was this done in the two way ANOVA analyses? Please comment on this.

A 2-way ANOVA was performed with time, sex, time x sex as the fixed effects and each mouse as the random effect. This was followed up with Post hoc and Fisher’s LSD for additional analysis. A clarification was added to the statistics section within the methods to further explain. Thank you. Please see line 139-142.

Third interesting aspect that was not addressed. Phylum Verrucomicrobiota was higher in males after treatment which was not observed in females. The differential abundance analyses at genus-level did not show what genera were responsible for this. Please discuss why. Further, in humans Verrucomicrobiota (especially genus Akkermansia) are known to be involved in glucose metabolism and obesity. Also, genus Akkermansia is higher in females. This might be interesting in combination with the phenotype of these mice. If the number of words allows, I would like to see some comments on that in the discussion.

Akkermansia was the genus of Verrucomicrobiota found in this study. This information was added to the discussion lines 232-235. Thank you.

Analyses have been performed either at phylum-level or at genus-level, if I am correct. It is not clear how taxa were aggregated to these higher levels and it is not clear at what levels the analyses were performed, i.e., Bray-Curtis dissimilarity and differential abundances. Also, in lines 188 and 189, ASV-level analyses were mentioned but ASVs are basically at strain-level. Please make clear.

Thank you for identifying this confusion, it was clarified within the methods section.

Line 115, please indicate if bead beating was part of the protocol.

A clarification has been added on line 118, thank you.

Line 125, the protocol in the reference is not the manufacturer’s guideline. Illumina has another protocol. Please make sure the correct reference is given.

An additional reference has been added.

In line 140 it is stated that normality was confirmed, but most ASVs are not normally distributed. Please explain.

This statement was removed, it was in regard to additional data that was previously removed upon last reviewers request.

In line 141, I assume that “both p values and FDR-corrected p values <0.05 were considered significant”.

The statement within the methods was revised.

Throughout the text, taxonomic levels are written in plural form which is not always correct, e.g., phyla instead of phylum (lines 146, 149) and genera instead of genus (line 234).

These grammatical errors were corrected.

Sentence in line 138 is unclear.

This sentence was edited to make it clearer.

Reviewer #4: In this article, the authors investigated the effects of Fenbendazole on the gut microbiome of mice with hypertension and reported the difference in gut microbiome responses to the treatment between male and female mice.

This article reported an observation without controls over baseline status or any confounding factors, such as BMI, in female and male mice. The main conclusion “there are changes in the gut microbial communities after FBZ use in a sex dependent manner” is not very convincing with current study design or evidence present in the article.

Major issues:
1. Difference between baseline microbiome in male and female mice
According to figure2, there are sex-related differences at baseline. Microbiome components interact between each other. The observation of change in one single component might have multiple components involved. Plus the regression toward the mean effects in statistics. If the baseline microbiome was different between sex, then we will expect the change will be different. The authors need to calibrate for the baseline difference.

A clarification and a previous references were added to note that there were no difference between male and females prior to treatment. The main difference observed was in Verrucomicrobia post treatment specifically Akkermansia, which is clarified in the discussion.

2. Lack of controls over confounding factors
As mentioned by the authors, obesity has a great influence on gut microbiome and the treatment may have sex-dependent effects on obesity. Is there a difference in BMI between female and male mice at baseline? Is there a difference in the change of BMI between female and male mice during treatment? These were not reported.

One of the reviewers on the initial submission requested that we remove this information. Therefore, we will keep it omitted at this time.

3. Statistical analysis
This study collected fecal samples at two time points from each mice. Using baseline microbiome as reference and comparing within subject changes will be a better choice than current methods, for example difference in difference model. The authors may also consider propensity score matching to remove effects of confounding factors.

Based on comments from reviewer 3, we used a mixed ANOVA with repeated measures for the analysis, no difference was found between sex pre-treatment.

4. More details in “Changes at the ASV level with Fenbendazole treatment and phenotypic outcomes”
“Changes at the ASV level with Fenbendazole treatment and phenotypic outcomes” worth more emphasis than “Changes at the phyla level in BPH/5 mice with Fenbendazole treatment”. ASV level is more informative than phyla level in showing microbiome functions.

More detail was added to this section of the results, specifically the genera involved in the changes in Actinobacteria and Verrucomicrobia.

Minor issues:
1. “Treatment had a significant effect on community composition using PERMANOVA of the Bray-Curtis dissimilarity matrix, with both BPH/5 female and male microbial communities differing post-treatment but not different by sex or interaction (treatment p=0.002, sex p=0.3, interaction p=0.05) (Figure 3b).” For “ sex p=0.3”, what was compared? Female and male samples with both time points pooled together? This analysis is actually showing that treatment had much bigger effects than sex, not supporting the conclusion.

This was clarified in the last paragraph of the discussion, lines 271-273.

2. The resolution of figures is too low.
